# Electronic cigarette use and subjective cognitive complaints in adults

**DOI:** 10.1371/journal.pone.0241599

**Published:** 2020-11-02

**Authors:** Zidian Xie, Deborah J. Ossip, Irfan Rahman, Richard J. O’Connor, Dongmei Li

**Affiliations:** 1 Department of Clinical & Translational Research, University of Rochester Medical Center, Rochester, New York, United States of America; 2 Department of Public Health Sciences, University of Rochester Medical Center, Rochester, New York, United States of America; 3 Department of Environmental Medicine, University of Rochester Medical Center, Rochester, New York, United States of America; 4 Department of Health Behavior, Roswell Park Comprehensive Cancer Center, Buffalo, New York, United States of America; Universidad Adolfo Ibanez, CHILE

## Abstract

**Introduction:**

Electronic cigarette use (vaping) has become popular in recent years. The number of Americans with a variety of cognitive deficits has been increasing dramatically. This study aimed to examine the potential association of vaping with subjective cognitive complaints in US adults.

**Methods:**

A combined 2016 and 2017 Behavioral Risk Factor Surveillance System (BRFSS) national survey dataset yielded 886,603 adults who indicated their smoking and vaping status, as well as whether they had subjective cognitive complaints. With this dataset, the cross-sectional association of electronic cigarette use with subjective cognitive complaints was examined using multivariable weighted logistic regression models.

**Results:**

Both dual users (adjusted Odds Ratio [aOR] = 2.07; 95% Confidence Interval [CI] = 1.66 to 2.60) and current vapers who were either ex-smokers (aOR = 1.94; 95% CI = 1.40 to 2.71) or never smoked (aOR = 1.96; 95% CI = 1.16 to 3.30) showed a significantly higher association with subjective cognitive complaints than never users. Current smokers (aOR = 1.49; 95% CI = 1.32 to 1.69) and ex-smokers (aOR = 1.25; 95% CI = 1.11 to 1.41) had a significantly higher association with subjective cognitive complaints compared to never users. Compared to current smokers, the ex-smokers showed a lower association with subjective cognitive complaints (aOR = 0.84; 95% CI = 0.73 to 0.96). Finally, the association of vaping with subjective cognitive complaints was not statistically significant in individual age group.

**Conclusion:**

Similar to smoking, vaping is associated with subjective cognitive complaints in US adults. These results provide preliminary evidence for a cross-sectional association of vaping with potential cognitive health effects in adults.

## Introduction

Since introduced to the United States in 2007, e-cigarettes have become increasingly popular, and the use has skyrocketed among youth since 2016. While the prevalence of e-cigarette use remained stable or decreased in older age groups (age 25 and older), it increased among young adults (age 18–24) in the United States from 2014 to 2018 [1]. Based on the 2019 National Youth Tobacco Survey, it was estimated that in 2019 about 27.5% of high school students and over 10% of middle school students have used e-cigarettes in the United States [2]. E-cigarette use is suggested to be associated with several respiratory disorders (such as wheezing and chronic obstructive pulmonary disease) in adults [3–5], but its association with other health risks is largely unknown.

Cognitive deficits encompass impairments in information processing, which can lead to difficulty paying attention, processing, and responding to information quickly, recalling information, initiating speech, reasoning, and making judgments [6]. Estimates indicate that in 2000, more than 4.5 million people had Alzheimer’s disease (the most well-known form of cognitive impairment) in the United States, which will increase by 3-fold by 2050 [7]. Cigarette smoking has been linked with risk for Alzheimer’s disease [8,9]. In addition, a twin study showed that cigarette smoking during adolescence and adulthood significantly increased the risk of cognitive deficits (attention problems) [10]. Prospective clinical human studies showed that prenatal maternal smoking is associated with cognitive deficits in the offspring, including general intelligence, auditory functioning, and hyperactivity [11–13]. Through cross-sectional and longitudinal analyses, several prospective studies showed that young and middle-aged adults who currently smoke had a significantly higher risk of cognitive impairments based on neurocognitive assessment, including memory, attention, and executive function deficits [14–16].

The prefrontal cortex (PFC) area in the brain is involved in cognitive functions (such as attention and impulse control), and its development and activities are affected by nicotine exposure during adolescence and early adulthood, which can cause long-lasting cognitive impairments [17,18]. It has been shown that while low doses of nicotine exposure might improve cognitive functions, higher doses of nicotine exposure could impair cognitive functions, which depends on the interaction of nicotinic receptor systems with neural systems underlying cognitive functions [19]. Considering that e-cigarettes deliver comparable nicotine to traditional combustible cigarettes [20], it is plausible to hypothesize that the e-cigarette use might be associated with some cognitive problems. Maternal e-cigarette exposure has been shown to be associated with cognitive deficits in mouse offspring, such as short-term memory impairment and hyperactivity [21]. To date, no study has examined the potential association of e-cigarettes with cognitive deficits in humans.

In this study, we used combined 2016 and 2017 Behavioral Risk Factor Surveillance System (BRFSS) national survey data for adult participants to investigate the cross-sectional association of e-cigarette use (vaping) with subjective cognitive complaints in US adults. Our statistical analyses indicate a possible cross-sectional association between vaping and subjective cognitive complaints in US adults.

## Methods

### Study participants

Administered by the Centers for Disease Control and Prevention (CDC), the BRFSS is an annual cross-sectional health-related telephone interview survey on health-related risk behaviors, chronic health conditions, and preventive service use among adults (18 years or older) in all 50 US states as well as the District of Columbia and three US territories [22]. The participants were recruited using random digit dialing techniques on both landlines and cell phones. Since both 2016 and 2017 survey data contain the same interview questions related to e-cigarettes, we combined 936,319 adult participants from 2016 and 2017 BRFSS data in our analysis, including 486,303 adults in 2016 and 450,016 adults in 2017.

### Vaping and smoking categories

To carefully consider the possible long-lasting effect of previous smoking on subjective cognitive complaints, we distinguished previous smokers from never smokers, and current vapers who never smoked from current vapers who were ex-smokers to determine the unique association of vaping with subjective cognitive complaints. First, we defined four smoking or vaping groups:

Current established smokers --- Have smoked at least 100 cigarettes in their entire life, and now smoke every day or some days.
Previous smokers --- Have smoked at least 100 cigarettes in their entire life, and now do not smoke cigarettes at all.
Never smokers --- Have smoked less than 100 cigarettes in their entire life, and now do not smoke cigarettes at all.
Current established vapers --- Currently using e-cigarettes every day or some days.

Based on the above four groups and past smoking experience of vapers, we further grouped the adult participants into six smoking and vaping categories:

1. Current smokers: Current established smokers who were not current established vapers.
2. Ex-smokers: Previous smokers who were not current established vapers.
3. Current vapers who were ex-smokers: Current established vapers who were ex-smokers.
4. Current vapers who never smoked: Current established vapers who were never smokers.
5. Dual users: Current established smokers who were also current established vapers.
6. Never users: Never smokers who also were not current established vapers.

### Outcome variable and covariates

The outcome variable is based on the survey question: “Because of a physical, mental, or emotional condition, do you have serious difficulty concentrating, remembering, or making decisions?”. Depending upon the responses, the outcome variable, subjective cognitive complaints, has two levels, “yes” or “no”. Only those participants who answered either “yes” or “no” to this question were included in our study. With the purposeful variable selection method [5,23], covariates selected and controlled for in our data analysis included age, sex, employment status, education level, income level, self-reported general health categories, mental health (including stress, depression, and problems with emotions), alcohol use, and cannabis use. Except for mental health and cannabis use, all other covariates are categorical variables.

### Statistical analysis

To determine the association of subjective cognitive complaints with each covariate, weighted frequency distributions were calculated. Multivariable weighted logistic regression models were used to determine the association of smoking and vaping status with the outcome variable, subjective cognitive complaints, after adjusting for the effects of those covariates. Considering the complex sampling design, the variable _LLCPWT as the final weight for each respondent was included in our statistical models. The stratification variable _STSTR and the clustering variable _PSU were also included in our models. The 2016 and 2017 weights were divided by 2 as the final weight for the combined 2016 and 2017 BRFSS data. To determine the association of vaping and smoking with subjective cognitive complaints, adjusted Odds Ratios (aORs) from multivariable weighted logistic regression models and their 95% Confidence Intervals (CIs) were used. To examine the age effect on the association of vaping with subjective cognitive complaints, we divided adults into five groups, including “18–24”, “25–34”, “35–49”, “50–64” and “65+”. All statistical analyses were conducted using PROC SURVEY procedures in SAS V9.4 (SAS Institute Inc., Cary, NC) taking the complex sampling design into account. The Taylor series linearization method was used to estimate the standard deviations. All tests were two-sided with a significance level of 5%.

## Results

### Demographic characteristics of adults with subjective cognitive complaints

Among the 936,319 adults in either the 2016 or 2017 BRFSS survey, we included in our analysis 886,603 adults (95%) who indicated their current smoking and vaping status, as well as whether they reported having serious difficulty concentrating, remembering, or making decisions (subjective cognitive complaints). As shown in Table 1, there was a similar prevalence of subjective cognitive complaints in the young (18–34) and middle-aged (35–64) adults (11.41% and 11.57%) while it was low in those 65+ (9.63%). The prevalence of subjective cognitive complaints was higher among females (12.24%) than males (9.85%). Adults who were unable to work had the highest percentage of subjective cognitive complaints (45.94%), followed by adults who were out of work for one year or more (24.73%) and adults who were out of work for less than one year (17.45%). The percentage of adults who had subjective cognitive complaints decreased as the education level increased, from 21.30% to 4.67%. With the increase in income, the percentage of adults who had subjective cognitive complaints decreased from 28.41% to 4.10%. Similarly, with better general health, the percentage of adults who had subjective cognitive complaints decreased from 44.56% to 3.58%. Adults who reported at least one drink of alcohol in the past 30 days had a lower percentage of subjective cognitive complaints than those who did not (8.93% vs. 13.51%). Compared to adults who did not have subjective cognitive complaints, those who had subjective cognitive complaints reported more days of cannabis use in the past 30 days on average, 2.75 days vs. 1.13 days. Adults who had subjective cognitive complaints had more days with mental health problems in the past 30 days than adults who did not on average, 12.99 days vs. 2.76 days. Compared to never users, dual users, smokers and vapers had a higher percentage of subjective cognitive complaints.

**Table 1:** Demographic characteristics of adult participants with subjective cognitive complaints.

Variables	Levels	N	Subjective cognitive complaints (% with 95% CI)		P-value
			Yes (n = 92,437)	No (n = 794,166)	
**Age (years)**					<.0001
	**18–34**	139,837	11.41 (11.19, 11.65)	88.58 (88.52, 88.65)	
	**35–64**	425,582	11.57 (11.42, 11.73)	88.43 (88.38, 88.47)	
	**65+**	310,652	9.63 (9.43, 9.83)	90.37 (90.34, 90.41)	
**Gender**					<.0001
	**Male**	387,402	9.85 (9.69, 10.01)	90.15 (90.11, 90.20)	
	**Female**	498,914	12.24 (12.08, 12.40)	87.76 (87.71, 87.81)	
**Employment**					<.0001
	**Employed for wages**	360,933	6.51 (6.37, 6.66)	93.49 (93.46, 93.52)	
	**Self-employed**	76,937	7.08 (6.77, 7.41)	92.92 (92.86, 92.97)	
	**Out of work for 1 year or more**	19,011	24.73 (23.94, 25.55)	75.27 (74.97, 75.57)	
	**Out of work for less than 1 year**	17,643	17.45 (16.76, 18.16)	82.55 (82.30, 82.80)	
	**A homemaker**	48,825	10.25 (9.85, 10.66)	89.75 (89.64, 89.86)	
	**A student**	23,400	10.52 (10.03, 11.04)	89.48 (89.35, 89.60)	
	**Retired**	268,501	9.23 (9.03, 9.43)	90.77 (90.74, 90.81)	
	**Unable to work**	65,524	45.94 (45.57, 46.31)	54.06 (53.76, 54.37)	
**Education**					<.0001
	**Did not graduate high school**	65,410	21.30 (20.94, 21.66)	78.70 (78.52, 78.88)	
	**Graduated high school**	243,508	12.82 (12.62, 13.03)	87.18 (87.11, 87.24)	
	**Attended college or technical school**	245,050	10.72 (10.53, 10.91)	89.28 (89.23, 89.34)	
	**Graduated from college or technical school**	330,082	4.67 (4.57, 4.78)	95.33 (95.31, 95.34)	
**Income**					<.0001
	**Less than $10,000**	36,270	28.41 (27.88, 28.94)	71.59 (71.29, 71.91)	
	**$10,000 to $19,999**	95,038	21.33 (21.02, 21.65)	78.67 (78.50, 78.83)	
	**$20,000 to $34,999**	149,290	13.66 (13.40, 13.94)	86.34 (86.25, 86.42)	
	**$35,000 to $74,999**	228,359	7.84 (7.66, 8.03)	92.16 (92.12, 92.20)	
	**$75,000 or more**	242,706	4.10 (3.94, 4.26)	95.90 (95.88, 95.92)	
**General Health**					<.0001
	**Excellent**	149,126	3.58 (3.39, 3.78)	96.42 (96.40, 96.44)	
	**Very good**	289,663	5.32 (5.17, 5.47)	94.68 (94.66, 94.71)	
	**Good**	277,362	10.37 (10.18, 10.56)	89.63 (89.58, 89.68)	
	**Fair**	121,039	24.55 (24.24, 24.87)	75.45 (75.30, 75.60)	
	**Poor**	47,271	44.56 (44.08, 45.05)	55.44 (55.10, 55.78)	
**During the past 30 days, on how many days did you use cannabis?**					<.0001
	**Mean (95% CI)**	167,037	2.75 (2.43, 3.08)	1.13 (1.06, 1.20)	
**Adults who reported having had at least one drink of alcohol in the past 30 days?**					<.0001
	**Yes**	454,146	8.93 (8.79, 9.08)	91.07 (91.03, 91.10)	
	**No**	421,147	13.51 (13.33, 13.68)	86.49 (86.43, 86.55)	
**For how many days during the past 30 days was your mental health not good?**					<.0001
	**Mean (95% CI)**	873,326	12.99 (12.82, 13.15)	2.76 (2.73, 2.79)	
**Smoking and Vaping Status**					<.0001
	**Dual users**	15,868	27.53 (26.88, 28.21)	72.47 (72.04, 72.90)	
	**Current smokers**	114,390	20.37 (20.07, 20.67)	79.63 (79.51, 79.76)	
	**Ex-smokers**	244,610	11.00 (10.80, 11.21)	89.00 (88.95, 89.05)	
	**Current vapers who were ex-smokers**	8,808	18.68 (17.50, 19.94)	81.32 (81.07, 81.57)	
	**Current vapers who never smoked**	3,879	16.16 (14.81, 17.65)	83.84 (83.47, 84.21)	
	**Never users**	499,048	8.02 (7.88, 8.17)	91.98 (91.94, 92.02)	

### Cross-sectional association of vaping and smoking with subjective cognitive complaints

To examine the potential association of vaping and smoking with subjective cognitive complaints in US adults, we calculated the adjusted Odds Ratios (aORs) using multivariable weighted logistic regression models. The calculated aORs of subjective cognitive complaints for different covariates were consistent with the prevalence of subjective cognitive complaints in different demographic variables in Table 1, for example, males had significantly lower aOR for subjective cognitive complaints than females (S1 Table). Compared to never users, after adjusting for the covariates (including age, gender, employment, education, income, general health, cannabis use, and mental health), all smoking and vaping categories showed significantly higher aORs for subjective cognitive complaints in all adults, ranging from 1.25 to 2.07 (Table 2). Dual users of combustible and electronic cigarettes showed a significantly higher association with subjective cognitive complaints than never users (aOR = 2.07; 95% CI = 1.66 to 2.60). Current vapers who were ex-smokers or never smokers had similar and significantly higher aORs for subjective cognitive complaints than never users, with aOR = 1.94 (95% CI = 1.40 to 2.71) and aOR = 1.96 (95% CI = 1.16 to 3.30) respectively. Although the point estimators of the association with subjective cognitive complaints for current smokers or ex-smokers were relatively lower than for dual users and current vapers, they were still significantly higher than never users, with aOR = 1.49 (95% CI = 1.32 to 1.69) and aOR = 1.25 (95% CI = 1.11 to 1.41) respectively.

**Table 2:** Associations of smoking and vaping category with subjective cognitive complaints.

Smoking and vaping category	Adjusted OR (95% CI)					
	All adults (n = 875,621)	Age: 18–24 (n = 49,792)	Age: 25–34 (n = 90,045)	Age: 35–49 (n = 160,201)	Age: 50–64 (n = 265,381)	Age: 65+ (n = 310,652)
**Never users**	Reference	Reference	Reference	Reference	Reference	Reference
**Dual users**	**2.07 (1.66, 2.60)**	**2.06 (1.08, 3.92)**	1.58 (0.98, 2.55)	**2.71 (1.78, 4.13)**	**2.31 (1.48, 3.60)**	1.69 (0.95, 2.99)
**Current exclusive smokers**	**1.49 (1.32, 1.69)**	1.57 (0.98, 2.51)	**1.86 (1.39, 2.50)**	**1.55 (1.18, 2.03)**	**1.49 (1.21, 1.84)**	1.09 (0.84, 1.42)
**Current vapers who were ex-smokers**	**1.94 (1.40, 2.71)**	1.52 (0.63, 3.68)	**2.55 (1.12, 5.82)**	1.44 (0.80, 2.62)	**2.61 (1.51, 4.49)**	1.63 (0.56, 4.73)
**Current vapers who never smoked**	**1.96 (1.16, 3.30)**	1.61 (0.90, 2.90)	1.55 (0.44, 5.47)	4.39 (0.70, 27.63)	1.84 (0.41, 8.15)	0.04 (0.01, 0.27)
**Ex-smokers**	**1.25 (1.11, 1.41)**	1.08 (0.59, 1.96)	**1.61 (1.12, 2.31)**	1.18 (0.89, 1.56)	**1.35 (1.11, 1.65)**	1.10 (0.89, 1.37)
						
**Current smokers**	Reference	Reference	Reference	Reference	Reference	Reference
**Ex-smokers**	0.84 (0.73, 0.96)	0.65 (0.35, 1.24)	0.88 (0.60, 1.28)	0.76 (0.57, 1.02)	0.92 (0.73, 1.14)	1.03 (0.79, 1.35)
**Dual users**	**1.39 (1.11, 1.75)**	1.32 (0.69, 2.54)	0.85 (0.53, 1.36)	**1.72 (1.13, 2.63)**	1.55 (0.99, 2.44)	1.56 (0.86, 2.81)
**Current vapers who were ex-smokers**	1.30 (0.93, 1.82)	1.01 (0.42, 2.42)	1.35 (0.59, 3.10)	0.93 (0.51, 1.70)	1.72 (0.99, 2.98)	1.50 (0.51, 4.43)
**Current vapers who never smoked**	1.31 (0.78, 2.21)	1.08 (0.56, 2.06)	0.82 (0.24, 2.82)	2.82 (0.44, 17.98)	1.24 (0.28, 5.47)	0.03 (0.01, 0.25)

As shown in Table 2, dual users showed a higher aOR (aOR = 1.39; 95% CI = 1.11 to 1.75) compared to current smokers, which suggests that vaping is associated with subjective cognitive complaints. In addition, compared to current smokers, current vapers who were either ex-smokers or never smoked showed higher aOR that did not reach statistical significance, with aOR = 1.30 (95% CI = 0.93 to 1.82) and aOR = 1.31 (95% CI = 0.78 to 2.21) respectively, suggesting that vaping has a similar association with subjective cognitive complaints as smoking. Compared to current smokers, ex-smokers showed a lower aOR for subjective cognitive complaints (aOR = 0.84; 95% CI = 0.73 to 0.96), indicating that quitting smoking is associated with a lower risk of subjective cognitive complaints than continuous smoking.

### The association of vaping with subjective cognitive complaints by age group

Since the brain still undergoes significant development during adolescence and young adulthood, adolescent and young adults might be more susceptible to the neurobiological stimulus (such as nicotine) from vaping and smoking than older adults. To examine the age effect on the association of vaping with subjective cognitive complaints, we examined the association of vaping with subjective cognitive complaints in five age groups, 18–24, 25–34, 35–49, 50–64, and 65+ age groups (Table 2). While dual users and current exclusive smokers in most age groups showed significantly higher aORs for subjective cognitive complaints than never users, the point estimates of adjusted ORs of current vapers who never smoked in all five age groups are high but not statistically significant, for example, aOR = 1.61 (95% CI = 0.90 to 2.90) for 18–24 age group and aOR = 1.55 (95% CI = 0.44 to 5.47) for 25–34 age group (Table 2). However, by examining the number of subjects and subjects with subjective cognitive complaints in different smoking and vaping categories in different age groups (S2 Table), the sample size of current vapers who never smoked in several age groups was very small, for example, 67 in the 35–49 age group, 47 in the 50–64 age group, and 20 in the 65+ age group, indicating low power for these comparisons. Overall, with this dataset, we observed different associations of smoking and vaping with subjective cognitive complaints across different age groups in US adults.

## Discussion

Using the 2016 and 2017 BRFSS data, we investigated the cross-sectional association of smoking and vaping with subjective cognitive complaints in US adults. Compared to never users, current smokers had a significantly higher association with subjective cognitive complaints in adults, which is consistent with previous findings [10]. Notably, we also showed that current vapers who were either ex-smokers or never smokers had a similar and significantly higher association with subjective cognitive complaints than never users. Dual users of combustible cigarettes and e-cigarettes showed a significantly higher association with subjective cognitive complaints than never users. The association of vaping with subjective cognitive complaints was generally consistent with these results but not statistically significant when examined by age group, though cell sizes were small for some of these comparisons. Together, here we provide the first evidence that vaping is potentially associated with subjective cognitive complaints in US adults.

Compared to never users, ex-smokers had a significantly higher aOR in terms of subjective cognitive complaints. Our findings are in line with an animal study showing that previous nicotine exposure could impair attention in later life [17]. However, compared to current smokers, ex-smokers had a lower association with subjective cognitive complaints. One explanation for this observation is that the effects of smoking (eg. nicotine) on cognitive functions in the brain might attenuate after quitting smoking [24]. Another possible but less likely explanation is that ex-smokers might have quit smoking due to the improvement in their cognitive health problems.

Compared to combustible cigarettes, e-cigarettes contain fewer chemical constituents [25]. Therefore, e-cigarettes are considered to potentially have fewer adverse health effects than combustible cigarettes [26]. Several studies showed that compared to combustible cigarettes use, e-cigarette use had a lower association with risk of respiratory diseases, such as COPD and pneumonia [3,27,28]. Here, we compared the association with subjective cognitive complaints between vaping and smoking. Interestingly, current vapers who never smoked showed a relatively higher association with subjective cognitive complaints than current smokers even though the difference was not significant, suggesting that vaping at least has a similar association as smoking with subjective cognitive complaints, and the association of vaping with subjective cognitive complaints is independent of past smoking history. It has been demonstrated that nicotine plays a key role in the regulation of brain development [29]. Nicotine uptake of e-cigarette users could be similar to or even higher than that of cigarette smokers depending on the user behavior (such as puff duration) and the e-cigarette device [30,31], which might explain why vaping has a similar association with subjective cognitive complaints as smoking. The potential mechanism is that the chemicals contained in e-cigarettes (such as vegetable glycerin and propylene glycol, flavoring chemicals, nicotine) inhaled might be potentially translocated to the central nervous system like other ultrafine particles [32], where they could alter the central pacemaker within the suprachiasmatic nuclei in the hypothalamus, and therefore affect the cognitive functions. Our previous study showed that acute exposure to e-cigarettes could alter the expression of circadian molecular clock genes in mouse lungs [33]. Another possible explanation is that those who have cognitive health problems might use smoking or vaping to alleviate their cognitive problems.

Neurodevelopment continues through adolescence and extends into young adulthood, which makes the brains of youth or young adults more susceptible to the stimulus (such as nicotine) from combustible cigarettes or e-cigarettes [34,35]. Therefore, we examined whether the association of vaping with subjective cognitive complaints in adults is age-dependent. While current vapers who never smoked in most age groups except 65+ showed high aORs for subjective cognitive complaints, these associations were not statistically significant. Current vapers who never smoked in some age groups (for example, 35–49, 50–64, and 65+) had a relatively small sample size, which might result in the inconclusive results in these age groups. While current vapers who never smoked in two younger age groups (18–24 and 25–34) had a relatively large sample size, their aORs were still not statistically significant for subjective cognitive complaints. Several possible explanations include that these young adults have relatively shorter exposure time to e-cigarettes, or they are not susceptible to neurological impairments of vaping, or this could be due to the complex effects of nicotine on cognitive performance [36].

Current results could not determine the causal relationship between vaping and subjective cognitive complaints due to the cross-sectional characteristics of the BRFSS data. There are several possible different interpretations for the association of vaping with subjective cognitive complaints. One is that vaping or smoking could increase the risk of subjective cognitive complaints mainly through nicotine exposure. Another possible explanation is that patients having subjective cognitive complaints might use smoking or vaping to reduce cognitive symptoms. Several studies showed that mental health problems (such as anxiety, depressive, and substance use symptoms) could lead to the initiation of e-cigarette use [37–39]. One possible reason is that smokers or vapers believe smoking or vaping could help with their mental health problems [40]. However, due to a lack of information about the duration and frequency of vaping and subjective cognitive complaints in the BRFSS data, our results based on cross-sectional survey data could not support either explanation. Therefore, a longitudinal study is required to establish if vaping could increase the risk of subjective cognitive complaints.

In this study, our outcome measure, subjective cognitive complaints, is based on one survey question, “Do you have serious difficulty concentrating, remembering, or making decisions?”. Therefore, we did not directly measure the diagnosis of cognitive deficits. The responses to this question are relatively subjective, which could introduce some biases. This limitation might explain why the age group 65+ showed a lower percentage of subjective cognitive complaints than younger adults, which could also due to the complicated cognitive effects of nicotine [41]. Since this question contains several typical outcomes of cognitive deficits, including remembering, concentrating, or making decisions, we considered it a reasonable indicator for subjective cognitive complaints. In the future, to reliably measure cognitive deficits, a comprehensive and multi-item self-report tool designed to assess cognitive function, such as Self-Report Measure of Cognitive Abilities (SRMCA) [42], needs to be implemented.

Like any other survey studies, the BRFSS data might contain some recall bias, which might affect our results. However, considering its large sample size (nearly half a million subjects for the annual survey), this dataset more likely represents the US population than other surveys. Furthermore, previous evaluation of BRFSS data showed the high reliability of the BRFSS self-reported data [43]. Since the BRFSS data did not provide the information about the duration and frequency of vaping, as well as other important confounding variables (such as the quantity of other tobacco products use), we could not establish their effects on the association of vaping with subjective cognitive complaints, which might somewhat affect our results. Considering the short history of e-cigarettes in the market, we could not determine the long-term association of e-cigarette use with subjective cognitive complaints in this study. Therefore, our current results might underestimate the association of vaping with subjective cognitive complaints. To increase the sample size, we combined the 2016 and 2017 BRFSS survey data. While the overlapped participants between two surveys should be minimal considering the random selection of participants in each survey, these participants could introduce some bias in our data analysis.

Considering potential health risks associated with e-cigarettes, to combat the epidemic of e-cigarette use, since 2010 several states and local governments started to implement laws restricting the sale, marketing, and use of e-cigarettes [44], which has been shown to be associated with reduced e-cigarette use among US adults [45]. More recently, the US Food and Drug Administration (FDA) implemented the flavor enforcement policy on February 6, 2020, which restricts the sale of all flavored, cartridge-based e-cigarettes except tobacco and menthol flavors [46]. Furthermore, on May 18, 2020, New York State implemented the law to prohibit the sale of all flavored vapor products other than tobacco flavor [47]. While these laws/policies on e-cigarette use might allow us to better understand the association of e-cigarette use and cognitive problems, how they will affect the prevalence of cognitive deficits need further investigation. While the association of e-cigarette use with other health outcomes (such as respiratory symptoms/diseases, cardiovascular diseases), as well as the association of cigarette smoking with cognitive deficits, have been extensively studied, the potential association of e-cigarette use with cognitive problems is not well-studied. Using the national BRFSS survey data, we showed that similar to smokers, current vapers who were never smokers had a higher association with subjective cognitive complaints than never users in US adults. Furthermore, we showed that the association between vaping and subjective cognitive complaints was not statistically significant within each age group, which requires further investigation in the future. Together, in this study, we provided the very first evidence about the potential cross-sectional association of e-cigarette use with subjective cognitive complaints, which should raise concerns about possible cognitive effects of e-cigarette use, and further emphasize the importance of tobacco regulatory policy on flavored e-cigarettes to protect public health. Considering the popularity of e-cigarette use in adolescences, it will be critical to examine the potential association of e-cigarette use in youth with cognitive problems in the future.

## Supporting information

S1 Table The estimated adjusted odds ratios of subjective cognitive complaints for covariates. (DOCX)

S2 Table Sample size of smoking and vaping category with subjective cognitive complaints in five age groups. (DOCX)
