# Supplementary material for: Electronic cigarette use and subjective cognitive complaints in adults
Source: PLoS One. 2020 Nov 2;15(11):e0241599. doi: 10.1371/journal.pone.0241599 (PMC7605645; doi:10.1371/journal.pone.0241599)
Supplement: S1 Table — (DOCX) [file pone.0241599.s001.docx]

**S1 Table. The estimated adjusted odds ratios of subjective cognitive complaints** **for covariates**

| **Variables** | **Levels** | | | | | **aOR (95% CI)** |
| --- | --- | --- | --- | --- | --- | --- |
| **Age (years)** |  | | | | |  |
|  | **18-34** | | | | | 1.79 (1.48, 2.17) |
|  | **35-64** | | | | | 1.30 (1.12, 1.51) |
|  | **65+** | | | | | Reference |
| **Gender** |  | | | | |  |
|  | **Male** | | | | | 0.85 (0.77, 0.94) |
|  | **Female** | | | | | Reference |
| **Employment** |  | | | | |  |
|  | **Employed for wages** | | | | | 0.26 (0.22, 0.31) |
|  | **Self-employed** | | | | | 0.30 (0.24, 0.38) |
|  | **Out of work for 1 year or more** | | | | | 0.58 (0.46, 0.74) |
|  | **Out of work for less than 1 year** | | | | | 0.37 (0.28, 0.48) |
|  | **A homemaker** | | | | | 0.27 (0.22, 0.34) |
|  | **A student** | | | | | 0.34 (0.25, 0.47) |
|  | **Retired** | | | | | 0.49 (0.41, 0.59) |
|  | **Unable to work** | | | | | Reference |
| **Education** |  | | | | |  |
|  | **Did not graduate high school** | | | | | 1.64 (1.36, 1.99) |
|  | **Graduated high school** | | | | | 1.45 (1.27, 1.66) |
|  | **Attended college or technical school** | | | | | 1.36 (1.19, 1.55) |
|  | **Graduated from college or technical school** | | | | | Reference |
| **Income** |  | | | | |  |
|  | **Less than $10,000** | | | | | 2.20 (1.76, 2.76) |
|  | **$10,000 to $20,000** | | | | | 1.86 (1.53, 2.27) |
|  | **$20,000 to $35,000** | | | | | 1.54 (1.28, 1.86) |
|  | **$35,000 to $75,000** | | | | | 1.27 (1.07, 1.51) |
|  | **$75,000 or more** | | | | | Reference |
| **General Health** |  | | | | |  |
|  | **Excellent** | | | | | 0.24 (0.19, 0.32) |
|  | **Very good** | | | | | 0.35 (0.28, 0.42) |
|  | **Good** | | | | | 0.47 (0.40, 0.56) |
|  | **Fair** | | | | | 0.81 (0.68, 0.96) |
|  | **Poor** | | | | | Reference |
| **During the past 30 days, on how many days did you use cannabis?** | |  |  | |  |  |
|  |  | | | | | 1.02 (1.01, 1.03) |
| **For how many days during the past 30 days was your mental health not good?** | | | |  | |  |
|  |  | | | | | 1.08 (1.07, 1.08) |
