# Supplementary material for: Electronic cigarette use and subjective cognitive complaints in adults
Source: PLoS One. 2020 Nov 2;15(11):e0241599. doi: 10.1371/journal.pone.0241599 (PMC7605645; doi:10.1371/journal.pone.0241599)
Supplement: S2 Table — (DOCX) [file pone.0241599.s002.docx]

**S2 Table.** **Sample size of smoking and vaping category with subjective cognitive complaints**

| **Smoking and vaping category** | **# of subjects with cognitive complaints / # of subjects** | | | | | |
| --- | --- | --- | --- | --- | --- | --- |
|  | **All adults**  **(n = 875,621)** | **Age: 18-24**  **(n = 49,792)** | **Age: 25-34**  **(n = 90,045)** | **Age: 35-49**  **(n = 160,201)** | **Age: 50-64**  **(n = 265,381)** | **Age: 65+**  **(n = 310,652)** |
| **Never users** | 37,736/480,973 | 3,452/37,373 | 3,667/55,638 | 6,503/95,013 | 11,278/143,943 | 12,836/160,006 |
| **Dual users** | 4,363/15,778 | 499/1,743 | 797/3,062 | 1,243/4,023 | 1,438/5,127 | 386/1,823 |
| **Current exclusive smokers** | 22,420/113,633 | 1,160/4,966 | 3,049/15,616 | 5,688/26,469 | 9,314/42,151 | 3,209/24,431 |
| **Current vapers who were ex-smokers** | 1,607/8,751 | 157/812 | 290/1,746 | 419/2,368 | 575/2,618 | 166/1,207 |
| **Current vapers who never smoked** | 626/3,859 | 368/2,229 | 124/892 | 67/388 | 47/255 | 20/95 |
| **Ex-smokers** | 25,034/242,077 | 387/2,669 | 1,431/13,091 | 3,382/31,940 | 8,597/71,287 | 11,237/123,090 |
